# Effect of Cyclic Loading on Shear Bond Strength of Orthodontic Brackets: An In Vitro Study

**Published:** 2018-11

**Authors:** Mohammad Moslem Imani, Farzaneh Aghajani, Nafiseh Momeni, Mohammad Sadegh Ahmad Akhoundi

**Affiliations:** 1 Assistant Professor, Department of Orthodontics, School of Dentistry, Kermanshah University of Medical Sciences, Kermanshah, Iran; 2 Dental Research Center, Dentistry Research Institute, Department of Dental Biomaterials, School of Dentistry, Tehran University of Medical Sciences, Tehran, Iran; 3 Dentist, Dental Research Center, Dentistry Research Institute, Tehran University of Medical Sciences, Tehran, Iran; 4 Professor, Dental Research Center, Dentistry Research Institute, Department of Orthodontics, School of Dentistry, Tehran University of Medical Sciences, Tehran, Iran

**Keywords:** Shear Strength, Dental Bonding, Orthodontic Brackets, Bite Force

## Abstract

**Objectives:**

In clinical conditions, orthodontic brackets are exposed to periodic stresses mainly induced by mastication and intraoral forces. The objective of the present study was to evaluate the effects of cyclic loading to simulate masticatory forces on shear bond strength (SBS) of metal brackets bonded to teeth using self-etch and total-etch bonding systems.

**Materials and Methods:**

Eighty-four caries- and crack-free bovine mandibular incisors were selected and randomly assigned to two groups based on the type of bonding system. After bonding, all samples were thermocycled (500 cycles) followed by cyclic loading of the half of the specimens in each group by applying 40 N load with 2 Hz frequency for 10,000 cycles. The SBS was measured using a universal testing machine. The adhesive remnant index (ARI) score was calculated subsequently. Data were analyzed using Kolmogorov-Smirnov test, two-way ANOVA and Mann-Whitney test.

**Results:**

The SBS was 10.09±3.78 MPa and 14.44±6.06 MPa for self-etch and total-etch bonding systems in cyclic loading group, respectively. The SBS was 9.43±5.3 MPa and 11.31±5.42 MPa in self-etch and total-etch groups without cyclic loading, respectively. Cyclic loading did not cause any significant difference in SBS (P>0.05). The ARI scores of the groups were significantly different (P<0.05).

**Conclusions:**

The present results demonstrated that low masticatory forces at 10,000 cycles did not have a significant impact on bracket-adhesive SBS; however, they significantly changed the ARI score. Even though the total-etch bonding system yielded higher SBS than the self-etch system, the performance of both was clinically acceptable.

## INTRODUCTION

Optimal bracket-adhesive-enamel bond strength is essential for reliable transfer of forces from arch-wire to teeth. A number of factors, other than the bonding agents and brackets, can influence the bond strength of brackets to teeth. Among these factors, intraoral forces are of great significance [1]. Mastication, occlusion and orthodontic appliances produce dynamic stresses [2–4]. Although the magnitude of such cyclic stresses is lower than the static bond strength of brackets, they could compromise the bond strength and cause premature debonding of brackets during the treatment period. Therefore, in order to simulate the oral environment conditions in vitro, it is valuable to evaluate the effects of cyclic loading on bond strength [2, 5]. Orthodontists commonly use total-etch and self-etch bonding systems for bracket bonding to enamel surfaces [6]. In the etch and rinse systems, phosphoric acid is applied on the enamel surface as etchant. The enamel surface is then rinsed and dried before applying the adhesive. Self-etching adhesive systems are more recent and their manufacturers have combined the etching and priming steps into one step to decrease the chair time of bonding procedure [7]. These systems are less technique sensitive than total-etch systems due to the necessity of proper moisture control in the latter [8]. The etching pattern in self-etch bonding systems is similar to that in total-etch systems and provides acceptable bond strength [9,10]. However, during debonding procedures, self-etch bonding agents are less likely to cause enamel fracture [11]. Due to their periodic nature and creating fatigue, masticatory forces can affect the bond strength and/or mode of failure of bonded brackets, even when their magnitude is less than the bond strength [1–3]. Since there is limited understanding about the effects of cyclic loads on bond strength and mode of failure of bonded brackets, this study aimed to evaluate and compare the effect of cyclic loading on shear bond strength (SBS) of metal brackets bonded to enamel surfaces using total-etch and self-etch bonding systems.

## MATERIALS AND METHODS

In the present study, 84 extracted bovine mandibular incisors were selected and disinfected by immersion in 0.5% chloramine T solution at 4°C for one week after removing the soft tissue remnants and debridement. The teeth were caries-free and had no apparent defects or enamel damage. Although bovine and human teeth are different, studies have shown that there is no significant difference in adhesive bond strength to enamel between the two [12,13]. The roots of the teeth were cut, and the crowns were stored in distilled water at 4°C until testing. The teeth were randomly divided into two groups for use of total-etch (Transbond XT; 3M Unitek Orthodontic Products, Monrovia, CA, USA) and self-etch (Transbond Plus; 3M Unitek Orthodontic Products, Monrovia, CA, USA) bonding agents (TE and SE groups, respectively). Adhesive systems were applied according to the manufacturers’ instructions. Briefly, in the total-etch group, the teeth were etched with 37% phosphoric acid gel for 20 seconds and thoroughly rinsed with water for 15 seconds and dried with oil-free air spray for 2–5 seconds. Next, a thin layer of unfilled resin (Transbond XT) was applied with a micro-brush and dried for 2–5 seconds. In the self-etch group, the two components of Transbond Plus self-etching primer were mixed and applied on the labial surface of the teeth according to the manufacturer’s instructions followed by gentle air drying for 2–5 seconds.

### Bracket bonding:

Stainless-steel upper right central incisor brackets (Ultratrim Edgewise bracket; Dentaurum, Ispringen, Germany) with a base surface area of 12.68 mm^2^ were bonded to bovine incisors with Transbond XT light-cure composite (3M Unitek Orthodontic Products, Ontario, Canada) following the application of bonding agent. Before curing, excess composite resin was removed with an explorer and then the resin was cured for 20 seconds, 10 seconds mesially and 10 seconds distally, using a light-emitting diode light-curing unit (Ortholux LED; 3M Unitek Orthodontic Products, Ontario, Canada). All samples were prepared and bonded by one operator, and 300 g force was applied during bracket bonding using a force gauge (ZUG-UND 28, 450 g; Dentaurum, Ispringen, Germany). After bonding, all samples were stored in distilled water at 37°C for 24 hours. Subsequently, the samples were thermocycled for 500 cycles between 5°C and 55°C with a dwell time of 60 seconds. The transfer time between baths was 8 seconds.

### Shear bond strength testing:

After thermocycling, all samples were mounted in auto-polymerizing acrylic resin such that the bonding surface was perpendicular to the horizontal plane. For this purpose, after bonding of samples, a straight 25x21 stainless steel wire was placed in the bracket slot to ensure perpendicular position of bonded surface relative to the horizontal plane. Afterward, each group was divided into two subgroups; one subgroup was subjected to cyclic loading (10,000 cycles with 2 Hz frequency) [9,14, 15] with 40 N force (TE-C and SE-C groups), followed by SBS testing and the other subgroup was subjected to SBS testing without cycling loading (TE-N and SE-N groups). Selection of 40 N force was because of the fact that it is the lowest amount of force that is reportedly applied to orthodontic appliances during mastication [16]. The SBS of all samples was measured using a universal testing machine (Z050; Zwick Roell, Ulm, Germany) with a cross-head speed of 1.0 mm/minute. The SBS (MPa) was calculated by dividing the failure load (N) by the bracket base area (mm^2^).

### Adhesive remnant index (ARI) scoring:

Following debonding, the enamel surface of each tooth and the bracket base were inspected under a stereomicroscope (SMZ800; Nikon, Tokyo, Japan) at ×10 magnification.

The modified ARI scores [17] were calculated based on the amount of adhesive remaining on the bracket surface, which could be contributed to the mode of failure. The modified ARI scores ranged from 5 to 0, where “5” indicated 100% of adhesive remained on the bracket, “4” indicated 100%-75%, “3” indicated 75%-50%, “2” indicated 50%-25%, and ”1” indicated less than 25% of adhesive remained on the bracket surface. The score of “0” pointed to no adhesive remained on the bracket. Finally, two teeth from each group [18] were randomly selected, sputter-coated with gold and examined under a scanning electron microscope (SEM XL30; Philips International Inc., Potomac, MD, USA) operating at 15 kV to evaluate the differences in enamel surface quality among the groups.

### Statistical analysis:

Kolmogorov-Smirnov test confirmed normal distribution of data. Thus, the collected data were subjected to descriptive statistics. Two-way ANOVA was used to determine the effect of cyclic loading and adhesive system on SBS. The Mann-Whitney U test determined the differences in ARI scores among the groups. Significance for all statistical tests was determined at P<0.05, and SPSS version 15.0 was used to analyze the data.

## RESULTS

**Fig. 1.**
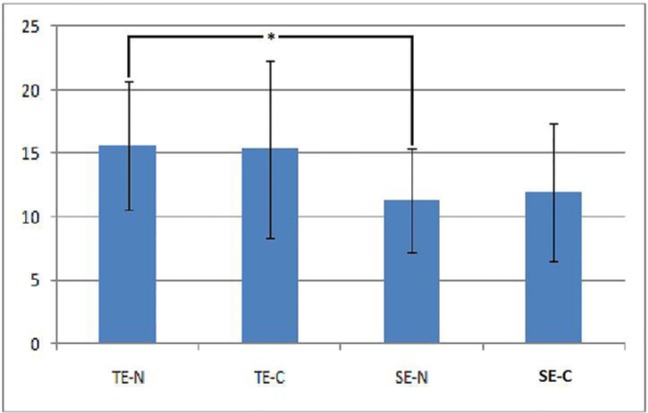
Shear bond strength of total-etch (TE) and self-etch (SE) with (-C) and without (-N) cyclic loading; ^*^ represents a significant difference (P=0.004).

Two-way ANOVA indicated that the effect of cyclic loading on SBS was not significant (P=0.1). The interaction effect of cyclic loading and the adhesive system on SBS was not significant either (P=0.28), but the type of adhesive system had a significant effect on SBS (P=0.008). The SBS was significantly higher in TE-N compared to SE-N group (15.61±5.01 and 11.29±4.02 MPa, respectively, P=0.004). In samples subjected to cyclic loading, the mean SBS was lower in SE-C group (11.92±5.39 MPa); however, this difference was not significant (P=0.101). Comparing SBS before and after cyclic loading revealed no significant difference (P=0.684 and P=0.877 in SE and TE groups, respectively). Figure 1 illustrates the results of the bond strength test.

The Mann-Whitney U test revealed a significant difference between the subgroups of each bonding agent in ARI score (P<0.0001); in SE-N group, more than 50% of samples had a score of 5, which indicates obvious fracture on the enamel surface while after cycling loading only 28.6% of samples had a score of 5.

**Fig. 2.**
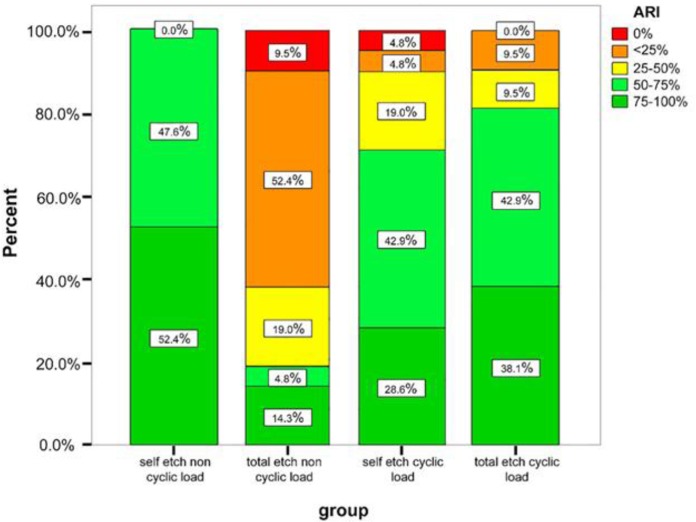
Adhesive remnant index (ARI) scores and their distribution in each group

**Fig. 3.**
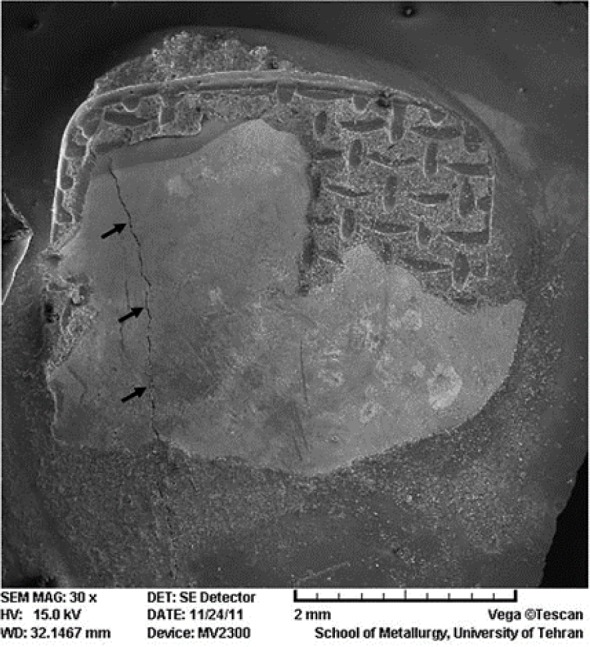
SEM image of an enamel surface of a specimen in the total-etch group. Arrows point to the enamel crack developed after debonding.

Opposite results were observed in the total-etch group; 52.4% of samples had a score of 1, which means most of the adhesive remained on the tooth surface (and less than 25% of adhesive remained on the bracket base); whereas, after cyclic loading, less than 5% of samples showed a score of 1. Comparing the ARI scores between TE-N and SE-N showed a significant difference between the two groups (P=0.002); however, after cyclic loading, the difference was not significant (P=0.44). Figure 2 illustrates the distribution of ARI scores. SEM assessment of specimens revealed presence of enamel cracks in one sample in total-etch group, which was not subjected to cyclic loading (Fig. 3).

## DISCUSSION

The aim of the present study was to evaluate the effect of cyclic loading on SBS of metal orthodontic brackets bonded to enamel surfaces with two commonly used adhesive systems.

In measuring the SBS of brackets to tooth structure, the blade technique has shown good reproducibility [19]. Moreover, it has been shown that forces applied to brackets during mastication are mainly of shear type [20]. Therefore, this method may be able to simulate the clinical setting [3]. However, Mojtahedzadeh et al. [21] showed that the wire loop method resulted in less dispersed SBS data. Since cyclic loading is similar to the blade technique, this method was used in our study. Despite the general assumption that cyclic loading would decrease the SBS [2, 9, 14, 22], the present results showed that SBS was not affected by cycling loading. In most studies in this field, the staircase or up and down method was employed to evaluate the effect of cyclic fatigue on bond strength [2, 9, 14, 22]. This method characterizes the total fatigue life of a material for a predefined number of cycles. The staircase method creates an experimental group for comparison with non-fatigued control group. In the present study, an effort was made to evaluate the effect of masticatory forces on bond strength of brackets to tooth surface in order to simulate intraoral conditions. In fatigue tests, cycles and loads would be applied such that they would increase the possibility of crack formation, and the results are presented as material durability. However, the present study aimed at finding whether intraoral (mastication) forces would affect the SBS of brackets.

In the present study, 40 N force was applied to bonded brackets since it is the lowest amount of force that is reportedly applied to orthodontic appliances during mastication [23]. Also, since most orthodontic patients are between early childhood and adolescence, 40 N seems more appropriate than higher values which have been reported for adult bite force.

Most similar studies determined fatigue strength by applying 1000 cycles and compared it to static SBS [2,3]. The amount of static bond strength was a basis for designing their staircase study regardless of the magnitude of masticatory forces or intraoral loads.

Although certain aspects of physical and mechanical properties of bonding systems can be clarified by in vitro experiments, it is necessary to simulate clinical conditions in laboratory testing as much as possible. In the current study, 10,000 cycles were applied because this was the highest frequency of cyclic loading submitted during fatigue testing [9, 14]. It has been reported that chewing and swallowing result in approximately 1,800 occlusal contacts per day [3]; however, not all occlusal contacts cause pressure on orthodontic brackets. In the present study, no significant differences were observed in either bonding group before and after cyclic loading. The lack of statistically significant differences in bond strength following cyclic loading might be because of an insufficient number of cycles to represent the clinical situation. In addition, the magnitude of chewing forces might be higher especially in young adults. Moseley et al. [24] reported that the effects of fatigue depend on the magnitude of load in cyclic loading.

The minimum acceptable bond strength of brackets is between 6–8 MPa [25]. Moreover, the bond strength should be less than the breaking strength of enamel, which is approximately 14 MPa [26]. Considering the results of the present study, the bond strength of both bonding systems was within the acceptable range. From a clinical point of view, it is important to choose an appropriate adhesive system, which withstands repeated intraoral forces and would not cause any damage to the teeth. Detecting enamel cracks in the TE-N group might raise some concerns; however, after cyclic loading, the ARI score significantly changed, and no enamel damage was observed.

The mean SBS of the self-etch adhesive system was lower than that of the total-etch group; nonetheless, it was still within the clinically acceptable range. There is some controversy in comparison of total-etch and self-etch adhesives. Although a number of studies have shown a higher value of SBS in total-etch groups [9,27], some studies found no significant difference between the two systems [26,28].

It has been shown that it is more desirable to have resin remnants on the enamel surface after bracket debonding to decrease the risk of enamel damage [29]. In this study, ARI scores showed significant differences between subgroups of both adhesive systems. In the TE-N group, a greater extent of adhesive remained on the tooth surface but in self-etch group with lower SBS, the majority of composite remained on the bracket surface. This finding is consistent with the statement that the amount of adhesive remnant tends to increase at high SBS [30]. However, other factors such as bracket retention might have a considerable effect on the ARI score [20].

In order to more realistically simulate the clinical conditions, further studies with higher load and higher frequency of cycles are required to provide more detailed information in this respect.

## CONCLUSION

The results of the present study demonstrated that low masticatory forces of 40 N at 10,000 cycles could not cause a significant difference in bracket-adhesive SBS; however, they could significantly change the ARI score. Although the mean SBS of total-etch system was higher than that of self-etch system, this difference was no longer significant after cyclic loading, and all values were within the clinically acceptable range. It seems that self-etch bonding systems are superior to total-etch systems for routine use in orthodontic clinics considering their optimal bond strength as well as other advantages of self-etch systems.
